# Presence of Activated (Phosphorylated) STAT3 in Radiation Necrosis Following Stereotactic Radiosurgery for Brain Metastases

**DOI:** 10.3390/ijms241814219

**Published:** 2023-09-18

**Authors:** Paola Anna Jablonska, Nuria Galán, Jennifer Barranco, Sergio Leon, Ramón Robledano, José Ignacio Echeveste, Alfonso Calvo, Javier Aristu, Diego Serrano

**Affiliations:** 1 Department of Radiation Oncology, Clinica Universidad de Navarra, 31008 Pamplona, Spain; 2 IDISNA and Program in Solid Tumors, Center for Applied Medical Research (CIMA), University of Navarra, 31008 Pamplona, Spainacalvo@unav.es (A.C.); dserrano@unav.es (D.S.); 3 Department of Pathological Anatomy, Clinica Universidad de Navarra, 31008 Pamplona, Spain; 4 Department of Pathology, Anatomy and Physiology, School of Medicine, University of Navarra, 31008 Pamplona, Spain; 5 CIBERONC, ISCIII, 28029 Madrid, Spain; 6 Department of Radiation Oncology and Proton Therapy Unit, Clinica Universidad de Navarra, 28027 Madrid, Spain; jjaristu@unav.es

**Keywords:** brain metastases, radiation necrosis, stereotactic radiosurgery, animal model, phospho-STAT3

## Abstract

Brain radiation necrosis (RN) is a subacute or late adverse event following radiotherapy, involving an exacerbated inflammatory response of the brain tissue. The risk of symptomatic RN associated with stereotactic radiosurgery (SRS) as part of the treatment of brain metastases (BMs) has been a subject of recent investigation. The activation of the signal transducer and activator of transcription 3 (STAT3) was shown in reactive astrocytes (RA) associated with BMs. Given that the pathophysiological mechanisms behind RN are not fully understood, we sought to investigate the role of STAT3 among other inflammatory markers in RN development. A mouse model of RN using clinical LINAC-based SRS was designed to induce brain necrosis with the administration of 50 Gy in a single fraction to the left hemisphere using a circular collimator of 5 mm diameter. Immunohistochemistry and multiplex staining for CD4, CD8, CD68, GFAP, and STAT3 were performed. For validation, eleven patients with BMs treated with SRS who developed symptomatic RN and required surgery were identified to perform staining for CD68, GFAP, and STAT3. In the mouse model, the RN and perinecrotic areas showed significantly higher staining for F4/80+ and GFAP+ cells, with a high infiltration of CD4 and CD8 T-lymphocytes, when compared to the non-irradiated cerebral hemisphere. A high number of GFAP+pSTAT3+ and F4/80+pSTAT3+ cells was found in the RN areas and the rest of the irradiated hemisphere. The analysis of human brain specimens showed that astrocytes and microglia were actively phosphorylating STAT3 in the areas of RN and gliosis. Phosphorylated STAT3 is highly expressed in the microglia and RA pertaining to the areas of brain RN. Targeting STAT3 via inhibition represents a promising strategy to ameliorate symptomatic RN in BM patients undergoing SRS.

## 1. Introduction

Brain metastases (BMs) are the most common form of intracranial tumor in the adult population and remain an important cause of morbidity and mortality. Owing to the advances in diagnostic neuroimaging and improvement in overall survival rates in the cancer patient population, the incidence of BMs is on the rise. Approximately 20–40% of cancer patients will develop BMs during their cancer journey, with the most common primary histologies including lung cancer (20–56%), breast carcinoma (20–30%), and melanoma (5–10%) [1]. BMs arise from the seeding of circulating tumor cells with the ability to penetrate through the blood–brain barrier (BBB) into the brain parenchyma, to subsequently colonize and adapt to the CNS microenvironment. This process involves complex molecular pathways and a selection of clones with specific functions that help evade the immune system.

BMs diagnosis requires early intervention and multidisciplinary management. Most common BMs-associated symptoms include headaches (50%), seizures (20%), focal neurological deficits (40%), gait disorders, and cognitive impairment [2]. Surgery is indicated in case of increased intracranial pressure and brain edema, usually followed by radiotherapy (RT) to the surgical bed. Administration of RT alone remains the cornerstone of BMs treatment and its use has increasingly moved towards more focal and accurate techniques, such as stereotactic radiosurgery (SRS) or hypofractionated stereotactic radiotherapy (HFSRT) [3,4]. SRS and HFSRT use high, ablative doses that are delivered to the target volume with a steep dose gradient, and result in local control rates as high as 70–90% [5].

However, both SRS and HFSRT are associated with a higher risk of radiation necrosis (RN). RN is a late adverse effect manifested by a massive neuronal cell death and extracellular edema. Symptomatic and asymptomatic RN cases have been reported in approximately 10% and 30% of BMs patients following SRS treatment [6], though the exact incidence varies, showing an increased trend with longer follow-up in BMs patients and concurrent administration of systemic therapies. Re-irradiation, dose prescription, and volume of BMs treated are the main risk factors associated with a higher incidence of RN [7].

The etiopathology of RN is not fully understood. Direct and indirect effects of ionizing radiation with high doses per fraction trigger a cascade of vascular, neuronal, and immunological mechanisms. Exacerbation of the inflammatory response is thought to represent the main process in the development of RN, and a deeper understanding is crucial to improve the existing therapeutic options and management strategies. Patients with a non-invasive diagnostic test that favors RN are initially managed with oral corticosteroids. Other alternatives include the administration of bevacizumab, a VEGF inhibitor, or hyperbaric oxygen therapy [8]. Despite the use of these therapies, RN may not be reversible. Mexiner et al. demonstrated that a recurrence of RN after initially improved or stable imaging occurred in 63% of patients [9]. The time to flare-up was longer after bevacizumab therapy than corticosteroids (median time of 5.6 (1–25.2) months versus 2.9 (0.3–24.1) months, respectively). Other authors question the clinical benefit of using anti-VEGF therapy once the radiation-induced damage has been established [10]. Surgery might be too morbid or simply not an option due to the RN location (i.e., the brainstem). Moreover, there is an ongoing debate whether concurrent administration of SRS/HFSRT and systemic agents such as targeted drugs or immunotherapy may increase the rates of symptomatic RN in these patients [11,12]. Therefore, additional therapeutic agents targeting the inflammation process are currently under investigation.

The signal transducer and activator of transcription 3 (STAT3) is a transcription factor involved in inflammatory responses, immune disorders, and carcinogenesis. STAT3 signaling plays a role in polarization of the macrophages into pro-inflammatory (M1) or anti-inflammatory (M2) types [13]. Moreover, STAT3 has been shown to be activated in reactive astrocytes (RA) associated with BMs [14]. RA also promote tumor growth and contribute to neurovascular dysfunction [15]. Prior reports described the presence of activated macrophages and RA in perinecrotic brain areas [16]. As the development of RN is highly influenced by immuno-inflammatory cascade and neurovascular alteration, we hypothesized that the phosphorylation of STAT3 plays a key role in the pathogenesis of RN following SRS/HFSRT treatment of BMs, and we aimed to test this hypothesis in an animal model, followed by validation in a series of human tissue specimens.

## 2. Results

### 2.1. Animal Model Design

We developed a mouse model of RN using clinical LINAC-based SRS to perform an in-depth characterization of the processes of immune cell infiltration and angiogenesis. For this purpose, we selected 10 Balb/c mice for brain SRS, and the irradiated brain hemispheres were compared to the contralateral, non-irradiated ones, to characterize radiation-induced changes using immunohistochemistry (IHC) and multispectral immunophenotyping. Five Balb/c mice did not undergo brain SRS and the non-irradiated brains were included as healthy controls. Mice were chosen as models because RN can be effectively induced and histopathological lesions resemble those found in patients, as observed in previously published studies [14,15].

### 2.2. Radiation Treatment Planning

**Figure 1:**
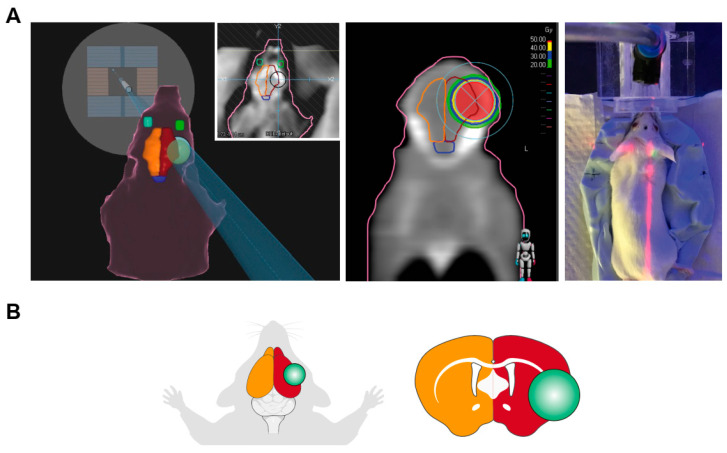
(**A**) Simulation and dosimetry planning of mouse SRS. Irradiated hemisphere is delineated in red, non-irradiated in yellow. The isocenter of the irradiated brain is represented by the green sphere. Anesthetized mice were placed in a silicone mold and aligned with lasers prior to the irradiation according to the simulation coordinates. A 5 mm circular collimator was placed in the left cerebral hemisphere, to allow for high dose sparing to the contralateral brain, brainstem, and the eyes. AP-PA field was used. Isodose line of 50 Gy is represented in red color. The figure in the middle shows the dose gradient and fall off isodose lines corresponding to 40 Gy, 30 Gy and 20 Gy. (**B**) Scheme of the irradiated areas and section of the brain showing the irradiated area (green circle).

An eight-week-old Balb/c mouse was anesthetized with isoflurane and placed in a silicon-based irradiation mold in a head-first prone position for immobilization and SRS planning. A high-resolution computed tomography (CT) (Siemens Somatom Emotion^®^, Munich, Germany) with 1.5 mm slice thickness was performed. The CT images were imported into Raystation^®^ Software (version 10.0) for SRS treatment planning using circular collimators. The isocenter was placed in a position posterior to the left eye, targeting the anterior part of the left cerebral hemisphere, ensuring dose sparing to the right hemisphere, eyes, and the brainstem. A 5 mm collimator was used, as LINAC-based SRS usually uses cone sizes of at least 4–5 mm diameter, which is comparable to the size of the mouse brain hemisphere. Two static fields at 0° and 180° using flattening filter-free 6 MV photon beams were generated to deliver a total dose of 50 Gy in 1 fraction. The total number of monitor units was calculated to deliver the prescribed dose to the isocenter. The prescription was chosen based on prior studies reporting the dose required for the induction of brain RN in mice and rats [17,18,19], and after carrying out a pilot dose escalation study at our institution, where no RN was observed within 3 months after applying a single fraction dose of 20 Gy, 30 Gy or 40 Gy. The treatment plan was subsequently exported to MOSAIQ^®^ (version 3.0) for treatment delivery using a linear accelerator (LINAC), Versa HD™. The dose distribution of the animal SRS treatment plan is shown in Figure 1.

### 2.3. Establishment of an Accurate Model of RN

**Figure 2:**
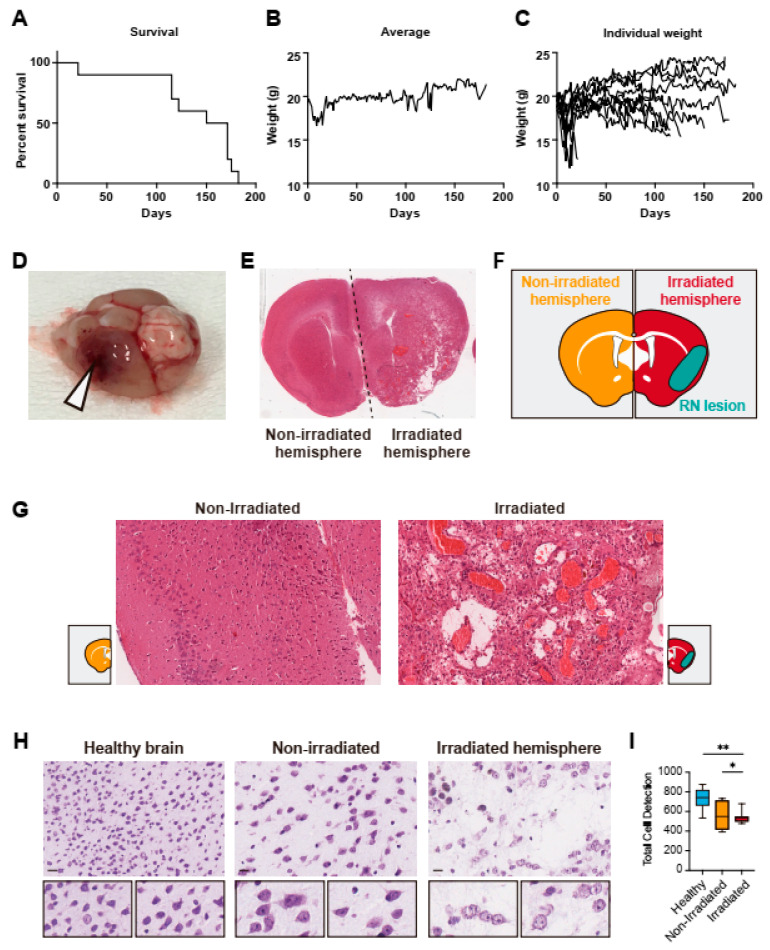
(**A**) Kaplan–Meier plot showing the survival of mice after the administration of 50 Gy to the left hemisphere of the brain to induce RN. (**B**) Average weight of animals after irradiation. Mice lost about 10% of weight in the first days after the irradiation, then recovered until RN was developed. (**C**) Individual values of mouse weights over the experimental period. Mice started to lose weight as a first sign of RN development. (**D**) Macroscopic observation of a dissected brain with a radionecrotic lesion (white arrowhead). RN lesions were highly vascularized and edematous. (**E**) Representative hematoxylin-eosin staining of a brain with a radionecrotic lesion in the irradiated hemisphere. Non-irradiated contralateral hemisphere had a normal aspect when observed under the microscope. (**F**) Scheme of the segmentation of non-irradiated and irradiated hemispheres as well as the radionecrotic lesion used for the histological analysis. (**G**) Detail of a characteristic H-E observation of non-irradiated hemisphere and radionecrotic lesion. Enlarged blood vessels are clearly observed. (**H**) Representative Nissl staining of healthy brain (control), non-irradiated hemisphere, and irradiated hemisphere. Scale bar corresponds to 20 µm. (**I**) Quantification of Nissl staining by QuPath revealed a significant neuronal loss in the irradiated brains compared to controls. *: *p* < 0.05; **: *p* < 0.01.

After irradiation, mice were daily monitored to assess whether they showed any behavioral changes. One of the mice died due to an acute brain injury 21 days after irradiation. A macroscopic observation of the brain revealed massive tissue destruction with extensive hemorrhage in both cerebral hemispheres. The rest of the animals started to develop RN lesions confirmed by histology in a range of 4 to 6 months after the irradiation of the brains with a single fraction of 50 Gy. All animals were sacrificed or died after 6 months post irradiation (Figure 2A). Since mice suffering any type of brain damage stop feeding, we also decided to monitor the weight of the animals. We noticed a significant weight loss in the first two weeks following irradiation (Figure 2B,C). Once recovered from the initial weight loss, animal weight remained stable or increased during the 4-month period until RN started to appear. In our experimental conditions, we observed that a sustained weight drop of approximately 15–20% was the first sign of RN development. No other behavioral changes were observed with the exception of variation in feeding habits.

A macroscopic examination of the brains revealed a congestive area compatible with RN, which corresponded to the SRS field (Figure 2D, see arrowhead). The non-irradiated contralateral hemisphere appeared unaltered. A hematoxylin-eosin histological analysis confirmed necrotic areas with loss of neuronal tissue in the irradiated hemisphere, but no changes in the contralateral hemisphere that served as control (Figure 2E). Next, we segmented the brains in three different regions for histological analysis: non-irradiated brain, radionecrotic area and irradiated brain (excluding the radionecrotic area, see Figure 2F). A histological analysis revealed that RN lesions were characterized by a structural disorganization of the brain and cellular loss, as well as increased vascularization, with a presence of aberrant blood vessels and areas with blood extravasation secondary to the destabilization of the blood–brain barrier (Figure 2G). Nissl staining revealed a significant (*p* < 0.01) neuronal loss in the irradiated brain hemisphere compared to the controls (Figure 2H,I).

### 2.4. Quantification of Immunohistochemical Changes in the Non-Neuronal Compartment and Activation of STAT3 in RN

**Figure 3:**
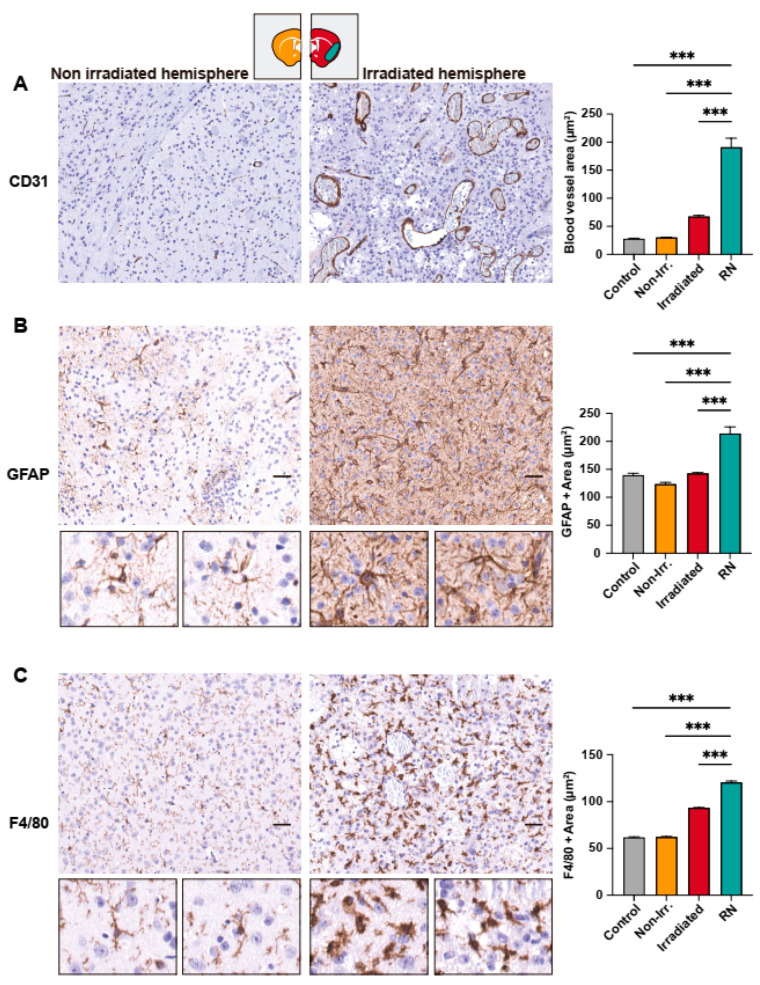
Immunohistochemical study of mouse brains. (**A**) Quantification of blood vessel area (measured as CD31 DAB stained area, in μm^2^) in non-irradiated and irradiated hemispheres of mice revealed a significant increase in the area of blood vessels in the RN region when compared to the unirradiated hemisphere and the control brain. (**B**) GFAP DAB staining area in the radionecrotic region is significantly higher (*p* < 0.001) compared to the rest of brain regions and healthy specimens. Insets show detail of astrocyte morphology when activated in the irradiated hemisphere. (**C**) Quantification of the F4/80 positive area in the radionecrotic region revealed a significantly higher area of microglia and macrophages when compared to other regions. Scale bar represents 50 µm. ***: *p* < 0.001.

For a more accurate measurement of changes in the vasculature, we used immunohistochemistry to detect CD31 in the mouse brain samples (Figure 3A). The representative IHC of control mice (non-irradiated) is shown in Supplementary Figure S1. The quantification of the blood vessels revealed an increased area of aberrant and congested capillaries within RN lesions when compared with the non-irradiated contralateral brain (*p* < 0.001) and the rest of the irradiated hemisphere (after excluding the RN area) (*p* < 0.001). These histological features have been previously described in patients with RN [20].

We subsequently assessed the alterations in the RN-surrounding microenvironment. For this purpose, IHC for the detection of GFAP (glial cells) and F4/80 (resident and circulant macrophages of microglia) was performed. QuPath analysis measured the DAB-positive stained area for GFAP and F4/80 in µm^2^. We found a higher area of GFAP+ cells (astrocytes) in RN lesions compared to the rest of the compartments (Figure 3B). Immunostaining for F4/80 also revealed a higher area of staining for microglia/macrophages in the entire irradiated hemisphere compared to the non-irradiated one (Figure 3C). Moreover, the RN lesions showed a significantly higher staining for F4/80+ (*p* < 0.001) than the irradiated hemisphere (Figure 3C). In both cases, the morphology of glial and microglial cells was different in the irradiated hemisphere. Cellular extensions in the cells surrounding the RN lesions were thicker and shorter, as revealed by GFAP+ and F4/80+ positive staining (see magnified details in Figure 3B,C). These changes suggest cell activation, as described during neuroinflammation, stress, and injury [21,22,23].

**Figure 4:**
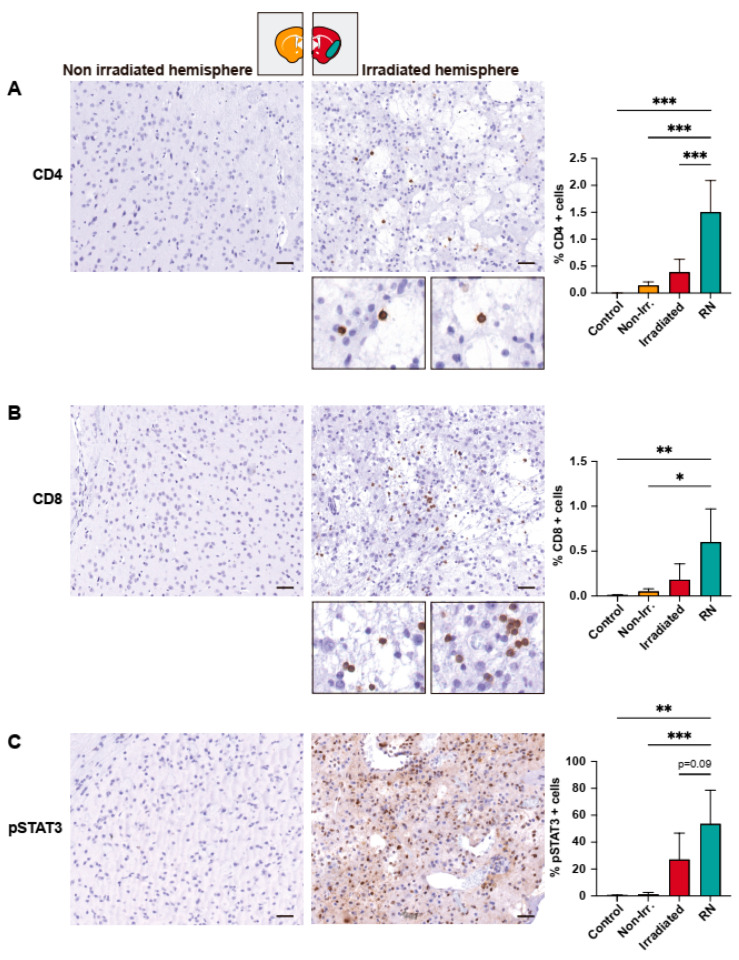
Immunohistochemical study of mouse brains. (**A**) Radionecrotic areas are characterized by a significant increase in the proportion of CD4+ lymphocytes. The irradiated area not corresponding to RN was also characterized by the presence of more CD4+ T cells than the non-irradiated area and the healthy control brain. (**B**) CD8+ infiltrate was also higher in the RN area compared to the non-irradiated hemisphere. (**C**) Phosphorylation of STAT3 was significantly increased in the RN area compared to the non-irradiated hemisphere of the brain and the healthy control brain. *: *p* < 0.05; **: *p* < 0.01; ***: *p* < 0.001.

Next, we evaluated whether the alteration of blood vessels, and the glial and microglial activation were able to elicit an immune response through the recruitment and infiltration by CD8 and CD4 T-lymphocytes in the RN areas. The quantification of CD4 and CD8 T-lymphocytes was performed with QuPath (as DAB-positive cells) and was expressed as the percentages of positive cells divided by the total of nucleated cells (hematoxylin-positive cells) in the segmented area (non-irradiated, irradiated, or RN). The quantification by IHC revealed a significant increase in CD4 T-lymphocyte infiltration in the RN area (irradiated hemisphere, *p* < 0.001; non-irradiated hemisphere, *p* < 0.001) (Figure 4A). Likewise, an increase in CD8 T-lymphocytes in the RN area compared to the non-irradiated hemisphere (*p* < 0.05) and to the irradiated hemisphere (*p* = 0.0563) was observed (Figure 4B). Subsequently, we analyzed the phosphorylation of STAT3 (Tyr705). As mentioned, STAT3 has been shown to be activated in RA in the presence of BMs [14] and astrogliosis [24]. In addition, STAT3 activation by phosphorylation controls lymphocyte T and B responses [25]. In the present study, we found that the percentage of pSTAT3 cells (expressed as the percentage of positive DAB-stained cells divided by the total of nucleated cells in the segmented area) was significantly higher in the RN area compared to the non-irradiated hemisphere (*p* < 0.001) and was also higher than that of the irradiated hemisphere (excluding the RN area), although in this case no statistical differences were found (Figure 4C).

**Figure 5:**
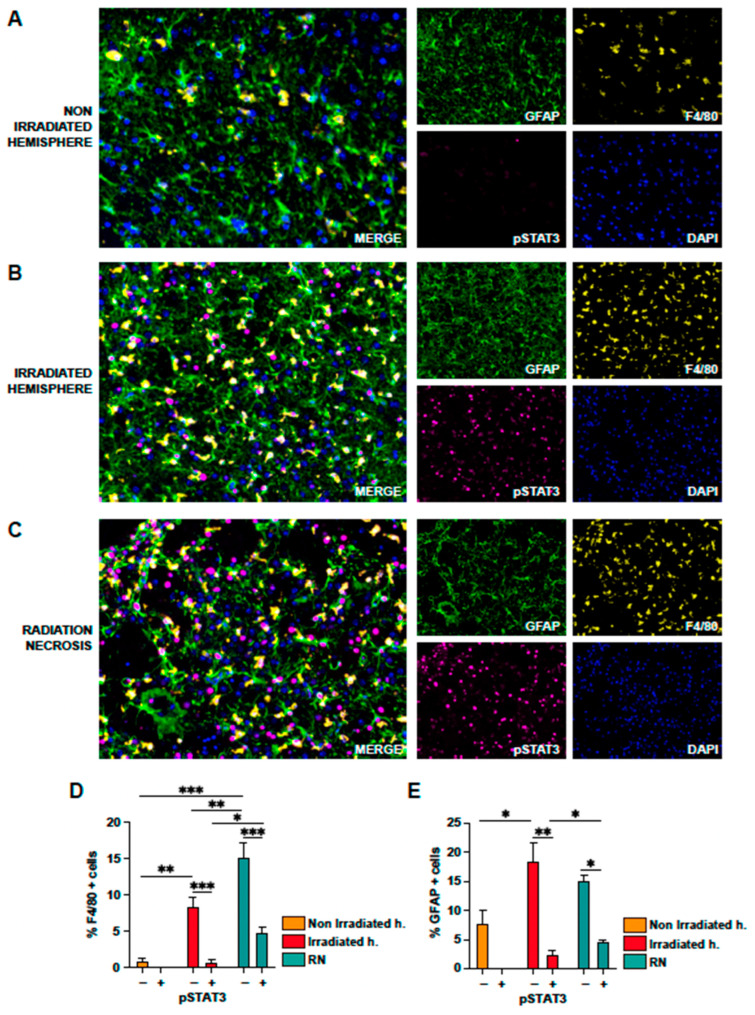
Multiplex immunofluorescence of irradiated and non-irradiated mouse brains. Detection of GFAP (green), F4/80 (yellow), pSTAT3 (magenta), and DAPI (blue) in brain samples from mice. Representative images of non-irradiated hemisphere (**A**), irradiated hemisphere (**B**), and radionecrotic lesion (**C**). (**D**) Quantification of microglia/macrophages (F4/80+ cells) and activated microglia/macrophages (F4/80+/pSTAT3+). (**E**) Quantification of astrocytes (GFAP+ cells) and reactive astrocytes (GFAP+/pSTAT3+). *: *p* < 0.05; **: *p* < 0.01; ***: *p* < 0.001.

To analyze in more depth the cell type where pSTAT3 was expressed, we performed multiplex immunofluorescence to study and quantify the presence of GFAP+/pSTAT3+ cells (RA) and F4/80+/pSTAT3+ (reactive microglia) in the non-irradiated hemisphere (Figure 5A), in the irradiated hemisphere (excluding the RN lesion, Figure 5B), and in the RN area (Figure 5C). Our study first confirmed a high number of GFAP+pSTAT3+ and F4/80+pSTAT3+ cells in the RN area and the rest of the irradiated area, compared to the non-irradiated hemisphere. In more detail, we detected that 15% of cells in the RN area corresponded to microglia and that approximately 30% of these microglial cells were reactive (F4/80+ pSTAT3+), whereas in the irradiated hemisphere (excluding the RN lesion) the percentage of reactive microglia was approximately 10% of microglial cells (Figure 5D). In the case of RA, we found that approximately 30% of astrocytes (GFAP+) had phosphorylated STAT3 in the RN area compared to 10% in the irradiated hemisphere (excluding the RN lesion, see Figure 5E).

### 2.5. Activation of STAT3 in Gliosis and Necrotic Areas of the Brain from Patients with Symptomatic RN

To answer the question whether pSTAT3 is activated in RA and microglia in human brain specimens, we identified a total of 11 patients with BMs, who underwent SRS and subsequently developed symptomatic RN or tumor recurrence, requiring brain surgery. Patient and treatment characteristics are presented in Table 1.

**Figure 6:**
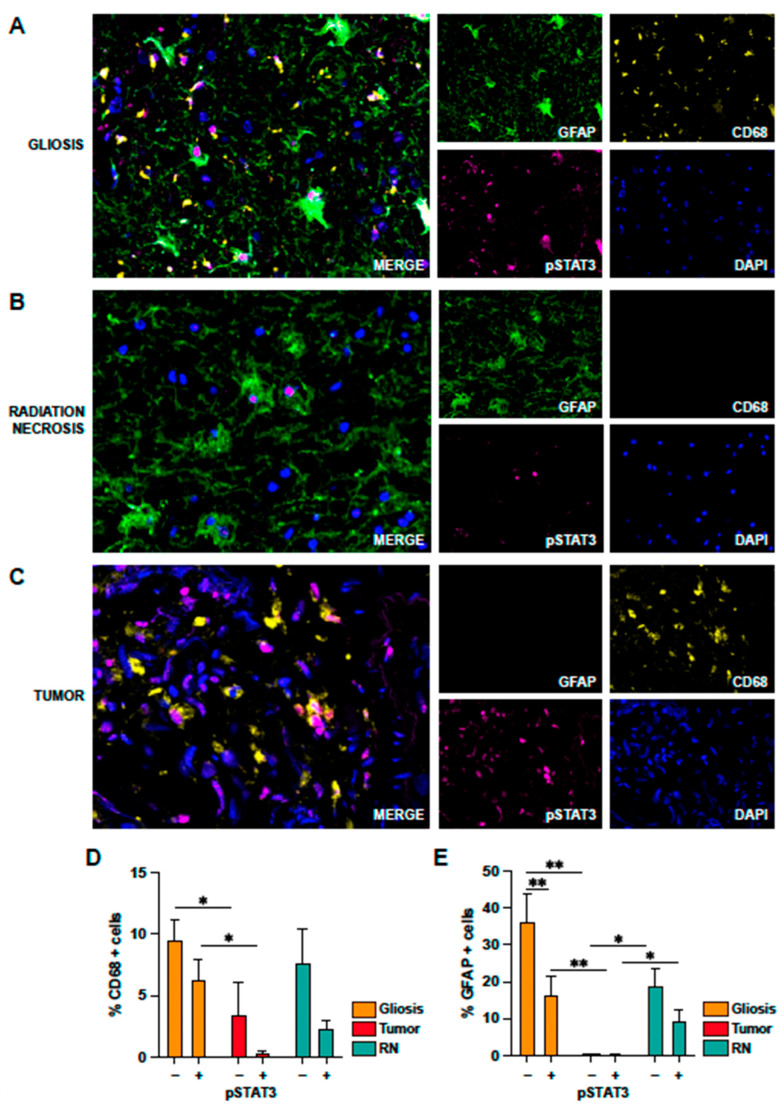
Multiplex immunofluorescence of human brain samples. Detection of GFAP (green), CD68 (yellow), pSTAT3 (magenta), and DAPI (blue) in brain samples from patients with symptomatic RN to study the activation of astrocytes (GFAP+/pSTAT3+) and microglia/macrophages (CD68+/pSTAT3+). Samples were segmented to identify areas of gliosis (**A**), radiation necrosis (**B**), and tumor (**C**). (**D**) Quantification of CD68 cells, represented as the percentage of CD68+ cells with respect to the total cells (nucleated cells in the segmented area) and double positive cells CD68+/pSTAT3+ (with respect to the total cells). These percentages were studied in the different regions delimited by the neuropathologist: gliosis, tumor, and radionecrotic lesion. (**E**) Percentage of astrocytes (GFAP+) with respect to the total cells (nucleated cells in the segmented area) and reactive astrocytes GFAP+/pSTAT3+ (with respect to the total cells). Percentages were determined in gliosis, tumor, and radionecrotic areas. *: *p* < 0.05; **: *p* < 0.01.

Using multiplex immunofluorescence, we found that astrocytes and microglia were actively phosphorylating STAT3 in the areas of gliosis (Figure 6A) and RN (Figure 6B). In the tumor areas, we solely detected CD68 corresponding to the infiltrating and activated macrophages (CD68+/pSTAT3+) (Figure 6C). After quantification of the multiplexed images, we encountered that 10% of total cells in the areas of gliosis corresponded to microglia and 35% to astrocytes (Figure 6D,E). Of these cells, approximately 66% of total CD68 cells (CD68+ pSTAT3+) and 45% of total astrocytes (GFAP+ pSTAT3+) were reactive. In the case of tumor areas, less than 5% of cells corresponded to infiltrating macrophages while astrocytes were mainly undetected. In the case of RN areas, we found that 8% of total cells corresponded to microglia and circulating macrophages and 20% to astrocytes. In the case of microglia, approximately 30% of CD68+ cells were also pSTAT3+ (reactive microglia) and half of the astrocytes were reactive (GFAP+ pSTAT3+).

## 3. Discussion

Symptomatic RN is a deleterious adverse effect of SRS/HFSRT following BMs treatment. In this view, there is a pressing need to better understand the underlying mechanisms of RN, and future strategies should focus on preventing RN development or its progression during the early stages. The design of animal models has become critical to decipher the biology of BMs seeding [26]. Specifically, animal models of brain RN following high-dose RT have been published, though the number of experimental studies in this field remains limited. Early experiments conducted in rats showed that the appearance of white matter necrosis was a function of the duration of RT exposure and cumulative dose [27]. The histopathological examination of rat brains radiated with 17.5, 20, 22.5, and 25 Gy in a single fraction showed demyelination and necrosis at 39 weeks post RT, with doses of 22.5 Gy or higher. The changes were seen sooner when 25 Gy were prescribed. Another model exploring the effects of a single fraction RT to rat spinal cords escalated the prescription from 8 Gy up to 22 Gy, detecting visible changes above the dose of 18 Gy [28]. In addition, the number of cells expressing HIF1α, and VEGF increased rapidly from 16 to 20 weeks post-radiation, and those cells were identified as astrocytes. Hartl and colleagues tested much higher radiation dose schemes to induce RN in a rat model [18]. They applied a single fraction dose ranging between 20 and 60 Gy, at 10 Gy intervals, delivered using a linear accelerator to the right brain hemisphere. In line with previously published work, they confirmed that the onset of RN was time- and dose-dependent, with 60 Gy leading to the fastest development of RN and doses below 30 Gy failing to induce RN in this model on subsequent MRI studies. A similar experimental study was designed using GK-SRS and a 4 mm collimator, to deliver 45 Gy, 50 Gy, and 60 Gy in one fraction to a single hemisphere in the brains of mice [17]. Around 4 weeks post-irradiation, MRI detected hyperintense regions corresponding to RN, with a significant expansion seen by 13 weeks. The time to RN onset was once again found to be related to the dose, with the pattern of injury being the same. From a histological standpoint, the areas of necrosis were represented by extensive tissue loss, edema, and fibrinoid vascular necrosis. Although histology was only acquired at a single time point—approximately at peak necrosis volume for each dose—the histological features did not significantly vary across the different doses. Finally, Devan et al. published a first rodent model of RN using clinical multiarc LINAC-based SRS and prescribing single doses of 60, 100, and 140 Gy. RN was induced after 8, 4–6, and 2 weeks, respectively, and MR findings correlated to the histopathological features of RN [19].

An interesting work by Yang et al. used a mouse model to further investigate the mechanism of hypoxia-induced inflammation in the development of late-onset radiation injury [29]. As previously shown, in addition to angiogenesis, an inflammatory response via the HIF1α and CXCR4 pathway has been hypothesized to contribute to the pathogenesis of brain RN. Following a single fraction of 50 Gy to one hemisphere using a GK-based SRS, mice were treated with either topotecan, a HIF1α inhibitor, or AMD3100, a CXCR4 antagonist. Follow-up imaging was carried out with MRI from 4 to 12 weeks after radiation. Conventional hematoxylin-eosin staining and immunohistochemistry staining were performed to assess the responses. In comparison with the control group, mice that were treated with topotecan or AMD3100 showed significantly smaller lesions on MRI and reduced tissue damage. Furthermore, the immunohistochemical analysis revealed lower expressions of VEGF, CXC chemokine ligand 12, CD68, CD3, and TNFα in the necrotic brain areas. Thus, it was concluded that both treatments were effective in reducing the inflammation to the brain after SRS, which was evidenced both radiologically and on the pathological specimens of RN. The authors suggested that anti-inflammatory agents could be combined with anti-angiogenic treatment as an adjuvant therapeutic strategy following RT for brain tumors, with additional benefits in the mitigation of RN development. In the same line, another preclinical study evaluated the effectiveness of a neuroprotective agent, SB415286, a specific inhibitor of the glycogen synthase kinase-3 beta (GSK-3β). GSK-3β is a multifunctional serine/threonine kinase abundantly expressed in the brain due to its vital role in neuronal signaling [30]. GSK-3β induces apoptosis in response to RT. This in vitro model aimed to investigate GSK-3β inhibition as a strategy of RN management. After GK irradiation with a single 45 Gy dose to the left hemisphere, mice were treated with SB415286 or DMSO (control group). The RN areas on MRI were significantly smaller at different post-RT time points in the intervention group. On histological examination, SB415286-treated animals had less tissue damage when compared to the classic RN features in the control group, including fibrinoid vascular necrosis, telangiectasia, and hemorrhage. These results, among other studies, show that exploring the mechanisms involved in the pathogenesis of RN may lead to adopting effective strategies that would help to mitigate or prevent the detrimental effects of RN development following RT to brain tumors, specifically BMs.

STAT3 is a key transcriptional regulator activated by phosphorylation of Tyr 705 or Ser 727 [31]. The JAK/STAT3 pathway is the canonical STAT3 signaling pathway, activated by cytokines or growth factors, transmitting signals to the nucleus to regulate downstream genes. In particular, interleukin-6 (IL-6) has been shown to play an important role in triggering STAT3 activation via JAK (Janus Kinase) stimulation in tumor cells and other tumor-infiltrating cell populations (i.e., immune cells), with a negative regulatory effect on dendritic cells, natural killer cells, effector T cells, and neutrophils, while positively regulating regulatory T cells and myeloid-derived suppressor cells [32,33]. STAT3 hyperactivation promotes several cancer-related processes, such as tumor-cell proliferation, survival, invasiveness, anti-apoptosis, angiogenesis, and formation of metastases [34].

STAT3 activation has been widely described in CNS and other malignancies, including hepatocellular, breast, or esophageal carcinoma [35,36,37]. This has led to the preclinical and clinical development of various IL-6/STAT3 inhibitors targeting the IL-6/JAK/STAT3 components in order to inhibit tumor growth and reduce immunosuppression in the tumor microenvironment [34]. For instance, Tong et al. demonstrated that STAT3 was a key component in the immunosuppression of high-grade glioma and could be inhibited by ACT001 [38]. A significant infiltration of immunosuppressive macrophages (M2 polarization) in these tumors could be reversed by targeting IL-6. Yang and colleagues demonstrated that the combination of IL-6 inhibition with CD40 stimulation reversed M2-mediated tumor immunosuppression, sensitized tumors to checkpoint blockade, and extended animal survival in two syngeneic GBM models [39]. Similarly, STAT3 activation was shown in RA involved in the formation of BMs exhibiting modulatory effects on the innate and acquired immune system that aid metastatic cell survival in the brain [13]. In addition to RA, activated microglia are widely present in the brain microenvironment, promoting BMs growth. Recently, a new approach for inhibiting BMs in NSCLC patients by targeting IL6/JAK2/STAT3 signaling in activated microglia was described by Jin et al. [40].

There is evidence of STAT3 pathway activation in the pathogenesis of radiation-induced injury following lung RT. In a mouse model, the protective effects of delayed treatment of WP1066 suggested that STAT3 signaling could be a therapeutic target for radiation pneumonitis [41]. However, to the best of our knowledge, the role of STAT3 in symptomatic RN following BMs treatment has not been previously described. In the present study, we designed a mouse model that resembled SRS/HFRST-induced brain RN, with the aim to identify the key elements of immune and inflammatory cell infiltration involved in the RN phenomenon and to explore the activation of the STAT3 signaling pathway. The feasibility of generating RN in mice using a clinical LINAC has been described previously [19]. We observed a significant damage to the endothelial cells both in the RN and perinecrotic areas, confirmed by CD31 positivity on the IHC and microscopic examination of the hematoxylin-eosin-stained brain samples. CD4 and CD8 infiltration was significant in RN areas, but also in the perinecrotic and surrounding brain tissue, showing strong immune activation in close vicinity to the RT target. CD68 and GFAP were predominantly infiltrating areas of RN, though similarly, the numbers were significantly higher in the totality of the irradiated hemisphere when compared to the non-irradiated one or the controls, indicating the activation of microglia and astrocytes in response to RT. Finally, pSTAT3 signaling was confirmed in the irradiated hemisphere and RN, substantiating our hypothesis regarding the activation of this pathway in the phenomenon of RN development upon SRS/HFSRT. These results warrant further preclinical studies with STAT3 as a potential target for symptomatic RN therapies.

To our knowledge, this is the first study that aimed to validate the preclinical findings in human tissue samples, with a segmentation of the different areas within each specimen that identified areas corresponding to RN and a perinecrotic zone corresponding to gliosis, residual or recurrent tumoral cells, hemorrhage, or normal brain parenchyma. We believe that such careful neuropathological examination is essential to explore the role of microenvironment cell infiltration and activation in symptomatic RN cases. Interestingly, we have found no GFAP and STAT3 positivity in the tumoral areas, though GFAP+ and GFAP+/STAT3+ were highly expressed in areas corresponding to RN and gliosis. This is consistent with the previously described fact of RA presence in the peritumoral zone of BMs, but not within the BMs itself. SRS/HFSRT treatment may favor a new recruitment of STAT3-mediated RA. The activation of microglia was confirmed predominantly in the RN areas, followed by tumor and gliosis. In the past, necrotic areas following RT for BMs were described as highly acellular and avascular; thus, this finding supports the fact that the process of symptomatic RN involves significant cell activation and infiltration. Future efforts of our group will focus on whether activated microglia represent M1 or M2 subtypes, in order to better understand if RN development maintains or reverses the immunosuppressed environment initially found in BMs.

Symptomatic RN involves an exacerbated response following high-dose RT, with endothelial and glial cell damage, leading to tissue hypoxia and initiating a cascade of inflammatory effects. Another interesting mechanism where STAT3 may play a role is neurovascular dysfunction and neuronal death. The participation of STAT3 in neuroinflammation and neurodegeneration (i.e., Alzheimer’s disease) has been confirmed in preclinical studies [42]. Also, inhibiting STAT3-mediated astrocyte reactivity could mitigate the cerebrovascular dysfunction associated with BMs [15]. Since targeting STAT3 may confer significant improvements in neurological outcomes for BMs patients, herein we propose that it may represent a valid therapeutic target in patients with symptomatic RN. Potential drugs targeting STAT3 could ameliorate the radiation-induced toxicity by acting upon the RA and microglia, as well as enhancing neuronal repair. STAT3 inhibition may improve the inflammatory RN environment by modulating the activation state of macrophages, inactivating the RA found in the perinecrotic areas, interrupting the RN-associated inflammatory and immune effects, and finally, hindering the propagation of neurovascular dysfunction.

The present study has its limitations. First, it involves a study of a small number of human RN samples. Multiplexed IHC used GFAP, CD68, and STAT3 staining selectively; it should be recognized that other cells and key mechanisms may be involved and should be explored in future studies. We carefully selected human RN samples from patients that did not have any exposure to immunotherapy or other specific agents in order to avoid additional bias during the brain sample analysis. However, nowadays most BMs patients receive targeted or immunotherapy agents, which may potentiate the RN-associated mechanisms. Therefore, future preclinical studies need a design that replicates current clinical patterns, where patients with BMs are concurrently treated with various systemic agents that cross the blood–brain barrier and thus exhibit an effect on the BMs and RN microenvironment. Finally, future efforts should focus on exploring the cytokines and other triggers activating the STAT3 pathway to design strategies that will prevent RN development at earlier stages.

## 4. Materials and Methods

### 4.1. Ethics Statement

All animal procedures were approved by the Institutional Animal Ethics Board and were conducted according to the ethical guidelines for the use of animals in research (reference number: 054-20). Patients signed an informed consent form to participate in this study, which was previously approved by the institutional ethics committee (reference number: 2019.086).

### 4.2. Treatment Delivery

Once anesthetized, each animal was immobilized using a previously confectioned silicone mold using positioning references and the source-to-surface distance of 97 cm. Mice were then irradiated under anesthesia with 2%/98% isoflurane/O_2_ as described, using a LINAC equipped with flattening-filter-free (FFF) 6 MV photons at a dose rate of 600 MU/min. The total radiation delivery time was under 25 min.

### 4.3. Post-SRS Follow Up, Study Endpoints, and Sample Collection

To prevent asthenia and weight loss after irradiation, mice nutrition was supplemented for a week with DietGel^®^ recovery (ClearH_2_O). The development and progression of necrosis was assessed daily by monitoring the weight loss and behavioral changes in mice. Animals were sacrificed if more than 20% of weight loss was observed over one week or when they showed stereotypies compatible with brain damage. The experiment was concluded at 6 months post irradiation, and at that time mice remaining alive were sacrificed. Brains were extracted and fixed in 4% formaldehyde at 4 ºC for 48 h, rinsed in 70% ethanol for 24 h, and embedded in paraffin for subsequent tissue histological processing.

### 4.4. IHC and Multispectral Immunophenotyping (Multiplex)

Paraffin-embedded 4 µm sections of the brain were used for IHC and multispectral immunophenotyping (hereinafter referred to as multiplex). Antibodies, antigen retrieval, and dilutions for the detection of CD4, CD8, F4/80, CD31, phospho-STAT3, and GFAP in brains by IHC are summarized in Supplementary Table S1. Primary antibodies were incubated with the EnVision+ System-HRP Labelled Polymer from Dako (ref. K4001 for mouse antibodies, ref. K4003 for rabbit antibodies). IHC was then developed with DAB+ (diaminobenzidine) Substrate chromogen system from Dako (ref. K3468). For quantification, slides were scanned with an Aperio CS2 scanner (Leica) and then the images were quantified and analyzed with QuPath 0.4.2. For analysis, brains were segmented in three different parts: RN area, irradiated hemisphere (excluding RN area), and non-irradiated hemisphere. Additionally, healthy brains were included as controls.

For multiplex, kits from Akoya Biosciences were used according to the manufacturer’s guidelines (NEL840001KT; OP7TL4001KT). Autofluorescence and positive control samples were included for validation purposes. Sample scanning and spectral unmixing of signals were conducted with the Vectra Polaris Automated Quantitative Pathology Imaging System (Akoya), using Phenochart 1.5 and InForm 2.5 softwares (Akoya). Image analysis and phenotyping were conducted using QuPath 0.4.2 [43]. Data were given as the number of cells with a specific immunophenotype divided by the total number of cells.

### 4.5. Analysis of Human Samples

We identified 11 patients who underwent SRS for BMs and subsequently developed symptomatic RN or tumor recurrence, requiring brain surgery. Hematoxylin-eosin-stained slides were prepared from formalin-fixed paraffin-embedded tissues by routine processing. Each sample was reviewed independently by two expert neuropathologists (R.R. and J.I.E.), to verify the pathological diagnosis and identify the areas of RN, tumor, gliosis (hypertrophy of glial cells), and normal brain parenchyma (see Supplementary Figure S2). In case of mixed pathology, if more than 25% of the tumor area was found in the surgical specimen, it was defined as tumor with areas of necrosis, whereas if there was less than 25% of tumor cells present, it was labeled as RN within tumor, as per prior reports [44,45].

Subsequently, multiplex was performed to detect microglia and circulating macrophages (CD68), and astrocytes (GFAP). Phosphorylation of STAT3 (Tyr705) was used to identify reactive astrocytes (RA) and microglia and to analyze the proportion of activated cells in the brain microenvironment and in the RN lesions.

### 4.6. Radiation Treatment Planning and Delivery

For SRS, patients were immobilized with a thermoplastic mask and an individual dental mold, as described in prior reports [46]. Briefly, radiation techniques (arch-therapy, IMRT, or VMAT) were selected depending on the location, shape, and size of the lesion, and delivered using a LINAC equipped with flattening-filter-free (FFF) X-rays, cone beam-CT, and robotic patient positioning platform (HexaPOD evo RT System). For treatment planning, a multiparametric magnetic resonance imaging (MRI) was obtained, unless contraindicated. A high-resolution CT simulation scan with 1.5 mm slice thickness without intravenous contrast was obtained. The brain CT-scan was rigidly fused with the multiparametric MRI using T1-weighted images. The gross tumor volume (GTV) was defined as the lesion visible on the T1-contrast-enhanced MRI. The PTV was created for each GTV by a 1.5–2 mm expansion. For SRS, a dose of 18 Gy was prescribed to 95% of the PTV. Lesions greater than 2.5 cm, previously treated with SRS or located less than 5 mm from the optic pathway or brainstem, were selected for 3- or 5-fraction treatment at a dose of 27 Gy or 32.5 Gy, respectively. Circular collimators were selected when the diameter of BM was less than 1 cm. Raystation v.10.0 software was used.

### 4.7. Follow-Up and Outcomes

Outcomes were assessed from the date of SRS. RN was determined based on MRI perfusion or defined as non-nodular tumor growth (including edema) that improved with steroids and became stable or smaller on follow-up MRI [47]. All RN events were graded using the Common Terminology Criteria for Adverse Events (CTCAE version 5.0). Local failure (LF) was defined by an increase in maximum tumor diameter or volume >10% on consecutive brain images [48]. BMs that appeared unchanged or smaller on follow-up MRI were considered controlled. In cases where it was unclear whether changes to the treated MBM represented LF versus RN, 1- or 2-month interval follow-up imaging was performed, using MRI perfusion sequences when available. If a consensus was still not reached, and the patient remained symptomatic after the follow-up imaging and ≥1 week of steroid treatment, surgical resection was recommended.

### 4.8. Statistical Analysis

Statistical analyses were conducted with GraphPad Prism 9.0 (GraphPad Software LCC). Normality of data was assessed using the Shapiro–Wilk test. Statistical differences for parametric analysis were determined with the Student’s *t* test or ANOVA (for comparison of more than two groups). Mann–Whitney *U* test or Kruskal–Wallis test was used to compare non-parametric experimental data of two groups or several groups, respectively. Values are expressed as mean ± SD, and statistical significance was defined as *p* < 0.05 (*), *p* < 0.01 (**) and *p* < 0.001 (***).

## 5. Conclusions

Activation of STAT3 is found in necrotic and perinecrotic areas of the brain in patients with symptomatic RN and in an animal model of RN. This may have future clinical implications. The delivery of SRS/HFSRT in combination with systemic agents, especially immunotherapy, may result in a hyperstimulation of the immune response, leading to an increased incidence of symptomatic RN in BMs patients. Thus, finding new immunomodulators and angiogenesis regulators that prevent RN development in this context is highly needed. The relevance of the present study relies on the fact that targeting the STAT3 pathway could ameliorate the risk of RN following BMs treatment. Subsequent studies should focus on evaluating the role of STAT3 inhibitors that cross the BBB to validate their potential as a therapeutic target.

## Figures and Tables

**Table 1:** Characteristics of patients who developed grade 3+ toxicity following SRS treatment. Surgical resection was performed, allowing for differential diagnosis between recurrent/residual tumor or radiation necrosis. None of the patients received immunotherapy. Three patients had prior brain RT (subject 5: WBRT, subject 8: SRS, subject 6: prophylactic WBRT) and one patient had subsequent brain RT (subject 9: WBRT with hippocampal avoidance). KPS: Karnofsky Performance Status; SRS: stereotactic radiosurgery; M: male; F: female; NSCLC: non-small cell lung cancer; SMALC: small cell anaplastic lung carcinoma; Gy: Grays. Age and KPS at the time of SRS treatment are provided.

ID	Age/Sex	KPS	Primary Diagnosis	SRS Dose and Location	Time to Post-SRS Surgery (Months)	Pathology Result	Microscopic Description (Tumor/Necrosis/ Gliosis/Normal Brain)	Systemic Therapy
1	45/F	80%	NSCLC	18 Gy/1 Right cerebellum	14 months	Radionecrosis within tumor	yes/yes/yes/yes	None
2	42/F	90%	NSCLC	16 Gy/1 Right frontal lobe	8 months	Radionecrosis within tumor	yes/yes/yes/no	Chemotherapy (Gemcitabine + Cisplatin)
3	74/M	80%	NSCLC	18 Gy/1 Right occipital lobe	6 months	Radionecrosis	yes/yes/yes/no	1st line: ALK-targeted therapy (Crizotinib) 2nd line: Immunotherapy (Pembrolizumab)
4	56/F	90%	NSCLC	16 Gy/1 Left frontoparietal lobe	42 months	Radionecrosis	yes/yes/no/no	Chemotherapy (Paclitaxel + Cisplatin)
5	67/F	90%	Breast ca	30 Gy/5 Left frontal lobe	18 months	Radionecrosis	yes/yes/yes/no	Chemotherapy (Paclitaxel)
6	60/M	90%	SMALC	16 Gy/1 Left frontoparietal lobe	5 months	Residual tumor	yes/no/yes/no	None
7	55/M	90%	NSCLC	16 Gy/1 Left occipital lobe	20 months	Recurrent tumor	yes/yes/no/no	Chemotherapy (Paclitaxel + Cisplatin)
8	49/F	80%	Breast ca	30 Gy/3 (reirradiation) Left cerebellum	18 months	Tumor with areas of necrosis	yes/yes/yes/yes	Chemotherapy + antiHer2 (Vinorelbine + Trastuzumab)
9	60/F	90%	Breast ca	18 Gy/1 Left temporal	20 months	Radionecrosis	yes/yes/yes/no	Chemotherapy (Docetaxel + Carboplatin)
10	65/F	90%	NSCLC	18 Gy/1 Left frontal lobe	6 months	Tumor with extensive areas of necrosis	yes/yes/yes/no	None
11	60/M	90%	NSCLC	18 Gy/1 Left frontal lobe	21 months	Radionecrosis	no/yes/yes/yes	None

## Data Availability

The datasets generated during and/or analyzed during the current study are available from the corresponding author on reasonable request.

## Supplementary Materials

The following supporting information can be downloaded at: https://www.mdpi.com/article/10.3390/ijms241814219/s1.

## References

[1] Fox B.D., Cheung V.J., Patel A.J., Suki D., Rao G. (2011). Epidemiology of Metastatic Brain Tumors. Neurosurg. Clin. N. Am..

[2] Clouston P.D., DeAngelis L.M., Posner J.B. (1992). The Spectrum of Neurological Disease in Patients with Systemic Cancer. Ann. Neurol..

[3] Lin J., Jandial R., Nesbit A., Badie B., Chen M. (2015). Current and Emerging Treatments for Brain Metastases. Oncology.

[4] Yamamoto M., Serizawa T., Higuchi Y., Sato Y., Kawagishi J., Yamanaka K., Shuto T., Akabane A., Jokura H., Yomo S. (2017). A Multi-Institutional Prospective Observational Study of Stereotactic Radiosurgery for Patients with Multiple Brain Metastases (JLGK0901 Study Update): Irradiation-Related Complications and Long-Term Maintenance of Mini-Mental State Examination Scores. Int. J. Radiat. Oncol. Biol. Phys..

[5] Vogelbaum M.A., Angelov L., Lee S.-Y., Li L., Barnett G.H., Suh J.H. (2006). Local Control of Brain Metastases by Stereotactic Radiosurgery in Relation to Dose to the Tumor Margin. J. Neurosurg..

[6] Jablonska P.A., Bosch-Barrera J., Serrano D., Valiente M., Calvo A., Aristu J. (2021). Challenges and Novel Opportunities of Radiation Therapy for Brain Metastases in Non-Small Cell Lung Cancer. Cancers.

[7] Kerschbaumer J., Demetz M., Krigers A., Nevinny-Stickel M., Thomé C., Freyschlag C.F. (2021). Risk Factors for Radiation Necrosis in Patients Undergoing Cranial Stereotactic Radiosurgery. Cancers.

[8] Vellayappan B., Lim-Fat M.J., Kotecha R., De Salles A., Fariselli L., Levivier M., Ma L., Paddick I., Pollock B.E., Regis J. (2023). A Systematic Review Informing The Management of Symptomatic Brain Radiation Necrosis after Stereotactic Radiosurgery and International Stereotactic Radiosurgery Society (ISRS) Recommendations. Int. J. Radiat. Oncol. Biol. Phys..

[9] Meixner E., Hörner-Rieber J., Lischalk J.W., Eichkorn T., Krämer A., Sandrini E., Paul A., Hoegen P., Deng M., Welzel T. (2023). Management of Initial and Recurrent Radiation-Induced Contrast Enhancements Following Radiotherapy for Brain Metastases: Clinical and Radiological Impact of Bevacizumab and Corticosteroids. Clin. Transl. Radiat. Oncol..

[10] Climans S.A., Ramos R.C., Jablonska P.A., Shultz D.B., Mason W.P. (2023). Bevacizumab for Cerebral Radionecrosis: A Single-Center Experience. Can. J. Neurol. Sci..

[11] Martin A.M., Cagney D.N., Catalano P.J., Alexander B.M., Redig A.J., Schoenfeld J.D., Aizer A.A. (2018). Immunotherapy and Symptomatic Radiation Necrosis in Patients with Brain Metastases Treated with Stereotactic Radiation. JAMA Oncol..

[12] Lehrer E.J., Kowalchuk R.O., Gurewitz J., Kondziolka D., Niranjan A., Lunsford L.D., Rusthoven C.G., Mathieu D., Malouff T.D., Bonney P. (2022). Concurrent Administration of Immune Checkpoint Inhibitors and Stereotactic Radiosurgery Is Not Associated with an Increased Risk of Radiation Necrosis: An International Multicenter Study of 657 Patients. Int. J. Radiat. Oncol. Biol. Phys..

[13] Xia T., Zhang M., Lei W., Yang R., Fu S., Fan Z., Yang Y., Zhang T. (2023). Advances in the Role of STAT3 in Macrophage Polarization. Front. Immunol..

[14] Priego N., Zhu L., Monteiro C., Mulders M., Wasilewski D., Bindeman W., Doglio L., Martínez L., Martínez-Saez E., Ramón Y. (2018). STAT3 Labels a Subpopulation of Reactive Astrocytes Required for Brain Metastasis. Nat. Med..

[15] Sarmiento Soto M., Larkin J.R., Martin C., Khrapitchev A.A., Maczka M., Economopoulos V., Scott H., Escartin C., Bonvento G., Serres S. (2020). STAT3-Mediated Astrocyte Reactivity Associated with Brain Metastasis Contributes to Neurovascular Dysfunction. Cancer Res..

[16] Furuse M., Nonoguchi N., Kawabata S., Miyatake S.-I., Kuroiwa T. (2015). Delayed Brain Radiation Necrosis: Pathological Review and New Molecular Targets for Treatment. Med. Mol. Morphol..

[17] Jiang X., Yuan L., Engelbach J.A., Cates J., Perez-Torres C.J., Gao F., Thotala D., Drzymala R.E., Schmidt R.E., Rich K.M. (2015). A Gamma-Knife-Enabled Mouse Model of Cerebral Single-Hemisphere Delayed Radiation Necrosis. PLoS ONE.

[18] Hartl B.A., Ma H.S.W., Hansen K.S., Perks J., Kent M.S., Fragoso R.C., Marcu L. (2017). The Effect of Radiation Dose on the Onset and Progression of Radiation-Induced Brain Necrosis in the Rat Model. Int. J. Radiat. Biol..

[19] Devan S.P., Luo G., Jiang X., Xie J., Dean D., Johnson L.S., Morales-Paliza M., Harmsen H., Xu J., Kirschner A.N. (2022). Rodent Model of Brain Radionecrosis Using Clinical LINAC-Based Stereotactic Radiosurgery. Adv. Radiat. Oncol..

[20] Vellayappan B., Tan C.L., Yong C., Khor L.K., Koh W.Y., Yeo T.T., Detsky J., Lo S., Sahgal A. (2018). Diagnosis and Management of Radiation Necrosis in Patients with Brain Metastases. Front. Oncol..

[21] Wilhelmsson U., Bushong E.A., Price D.L., Smarr B.L., Phung V., Terada M., Ellisman M.H., Pekny M. (2006). Redefining the Concept of Reactive Astrocytes as Cells That Remain within Their Unique Domains upon Reaction to Injury. Proc. Natl. Acad. Sci. USA.

[22] Bennett M.L., Viaene A.N. (2021). What Are Activated and Reactive Glia and What Is Their Role in Neurodegeneration?. Neurobiol. Dis..

[23] Woodburn S.C., Bollinger J.L., Wohleb E.S. (2021). The Semantics of Microglia Activation: Neuroinflammation, Homeostasis, and Stress. J. Neuroinflamm..

[24] O’Callaghan J.P., Kelly K.A., VanGilder R.L., Sofroniew M.V., Miller D.B. (2014). Early Activation of STAT3 Regulates Reactive Astrogliosis Induced by Diverse Forms of Neurotoxicity. PLoS ONE.

[25] Deenick E.K., Pelham S.J., Kane A., Ma C.S. (2018). Signal Transducer and Activator of Transcription 3 Control of Human T and B Cell Responses. Front. Immunol..

[26] Miarka L., Valiente M. (2021). Animal Models of Brain Metastasis. Neurooncol. Adv..

[27] Calvo W., Hopewell J.W., Reinhold H.S., Yeung T.K. (1988). Time- and Dose-Related Changes in the White Matter of the Rat Brain after Single Doses of X Rays. Br. J. Radiol..

[28] Nordal R.A., Nagy A., Pintilie M., Wong C.S. (2004). Hypoxia and Hypoxia-Inducible Factor-1 Target Genes in Central Nervous System Radiation Injury: A Role for Vascular Endothelial Growth Factor. Clin. Cancer Res..

[29] Yang R., Duan C., Yuan L., Engelbach J.A., Tsien C.I., Beeman S.C., Perez-Torres C.J., Ge X., Rich K.M., Ackerman J.J.H. (2018). Inhibitors of HIF-1α and CXCR4 Mitigate the Development of Radiation Necrosis in Mouse Brain. Int. J. Radiat. Oncol. Biol. Phys..

[30] Jiang X., Perez-Torres C.J., Thotala D., Engelbach J.A., Yuan L., Cates J., Gao F., Drzymala R.E., Rich K.M., Schmidt R.E. (2014). A GSK-3β Inhibitor Protects against Radiation Necrosis in Mouse Brain. Int. J. Radiat. Oncol. Biol. Phys..

[31] Cimica V., Chen H.-C., Iyer J.K., Reich N.C. (2011). Dynamics of the STAT3 Transcription Factor: Nuclear Import Dependent on Ran and Importin-β1. PLoS ONE.

[32] Yu H., Kortylewski M., Pardoll D. (2007). Crosstalk between Cancer and Immune Cells: Role of STAT3 in the Tumour Microenvironment. Nat. Rev. Immunol..

[33] Kortylewski M., Kujawski M., Wang T., Wei S., Zhang S., Pilon-Thomas S., Niu G., Kay H., Mulé J., Kerr W.G. (2005). Inhibiting Stat3 Signaling in the Hematopoietic System Elicits Multicomponent Antitumor Immunity. Nat. Med..

[34] Johnson D.E., O’Keefe R.A., Grandis J.R. (2018). Targeting the IL-6/JAK/STAT3 Signalling Axis in Cancer. Nat. Rev. Clin. Oncol..

[35] Xu J., Lin H., Wu G., Zhu M., Li M. (2021). IL-6/STAT3 Is a Promising Therapeutic Target for Hepatocellular Carcinoma. Front. Oncol..

[36] To S.Q., Dmello R.S., Richards A.K., Ernst M., Chand A.L. (2022). STAT3 Signaling in Breast Cancer: Multicellular Actions and Therapeutic Potential. Cancers.

[37] Ma R.-J., Ma C., Hu K., Zhao M.-M., Zhang N., Sun Z.-G. (2022). Molecular Mechanism, Regulation, and Therapeutic Targeting of the STAT3 Signaling Pathway in Esophageal Cancer (Review). Int. J. Oncol..

[38] Tong L., Li J., Li Q., Wang X., Medikonda R., Zhao T., Li T., Ma H., Yi L., Liu P. (2020). ACT001 Reduces the Expression of PD-L1 by Inhibiting the Phosphorylation of STAT3 in Glioblastoma. Theranostics.

[39] Yang F., He Z., Duan H., Zhang D., Li J., Yang H., Dorsey J.F., Zou W., Nabavizadeh S.A., Bagley S.J. (2021). Synergistic Immunotherapy of Glioblastoma by Dual Targeting of IL-6 and CD40. Nat. Commun..

[40] Jin Y., Kang Y., Wang M., Wu B., Su B., Yin H., Tang Y., Li Q., Wei W., Mei Q. (2022). Targeting Polarized Phenotype of Microglia via IL6/JAK2/STAT3 Signaling to Reduce NSCLC Brain Metastasis. Signal Transduct. Target. Ther..

[41] Yu J., Yuan X., Liu Y., Zhang K., Wang J., Zhang H., Liu F. (2016). Delayed Administration of WP1066, an STAT3 Inhibitor, Ameliorates Radiation-Induced Lung Injury in Mice. Lung.

[42] Toral-Rios D., Patiño-López G., Gómez-Lira G., Gutiérrez R., Becerril-Pérez F., Rosales-Córdova A., León-Contreras J.C., Hernández-Pando R., León-Rivera I., Soto-Cruz I. (2020). Activation of STAT3 Regulates Reactive Astrogliosis and Neuronal Death Induced by AβO Neurotoxicity. Int. J. Mol. Sci..

[43] Bankhead P., Loughrey M.B., Fernández J.A., Dombrowski Y., McArt D.G., Dunne P.D., McQuaid S., Gray R.T., Murray L.J., Coleman H.G. (2017). QuPath: Open Source Software for Digital Pathology Image Analysis. Sci. Rep..

[44] Detsky J.S., Keith J., Conklin J., Symons S., Myrehaug S., Sahgal A., Heyn C.C., Soliman H. (2017). Differentiating Radiation Necrosis from Tumor Progression in Brain Metastases Treated with Stereotactic Radiotherapy: Utility of Intravoxel Incoherent Motion Perfusion MRI and Correlation with Histopathology. J. Neurooncol..

[45] Tihan T., Barletta J., Parney I., Lamborn K., Sneed P.K., Chang S. (2006). Prognostic Value of Detecting Recurrent Glioblastoma Multiforme in Surgical Specimens from Patients after Radiotherapy: Should Pathology Evaluation Alter Treatment Decisions?. Hum. Pathol..

[46] Jablonska P.A., Serrano Tejero D., Calvo González A., Gimeno Morales M., Arbea Moreno L., Moreno-Jiménez M., García-Consuegra A., Martín Pastor S.M., Domínguez Echavarri P.D., Gil-Bazo I. (2020). Repeated Stereotactic Radiosurgery for Recurrent Brain Metastases: An Effective Strategy to Control Intracranial Oligometastatic Disease. Crit. Rev. Oncol. Hematol..

[47] Chao S.T., Ahluwalia M.S., Barnett G.H., Stevens G.H.J., Murphy E.S., Stockham A.L., Shiue K., Suh J.H. (2013). Challenges with the Diagnosis and Treatment of Cerebral Radiation Necrosis. Int. J. Radiat. Oncol. Biol. Phys..

[48] Snell J.W., Sheehan J., Stroila M., Steiner L. (2006). Assessment of Imaging Studies Used with Radiosurgery: A Volumetric Algorithm and an Estimation of Its Error. Technical Note. J. Neurosurg..

