# Supplementary material for: Presence of Activated (Phosphorylated) STAT3 in Radiation Necrosis Following Stereotactic Radiosurgery for Brain Metastases
Source: Int J Mol Sci. 2023 Sep 18;24(18):14219. doi: 10.3390/ijms241814219 (PMC10532304; doi:10.3390/ijms241814219)

Supplementary Materials

Supplementary table S1: List of antibodies and dilutions used in this study. N/a: non-applicable (not used in multiplex immunofluorescence).

**Antibody**	**Reference**	**Manufacturer**	**Dilution**	**Opal (Multiplex)**
CD4 (mouse, m)	25229	Cell signaling	1:100	n/a
CD8 (m)	98941	Cell signaling	1:500	n/a
F4/80 (m)	70076	Cell signaling	1:400	570
CD31 (m)	77699	Cell signaling	1:100	n/a
CD68 (human, h)	168M-94	Sigma-Aldrich	1:100	620
pSTAT3 (h/m)	9145	Cell signaling	1:100	620 (m), 480 (h)
GFAP (h/m)	3670	Cell signaling	1:200	520 (m), 570 (h)

Supplementary figure S1: Immunohistochemical study of mouse brains from the control group, showing CD31, CD4, CD8, GFAP, F4/80 and pSTAT3.

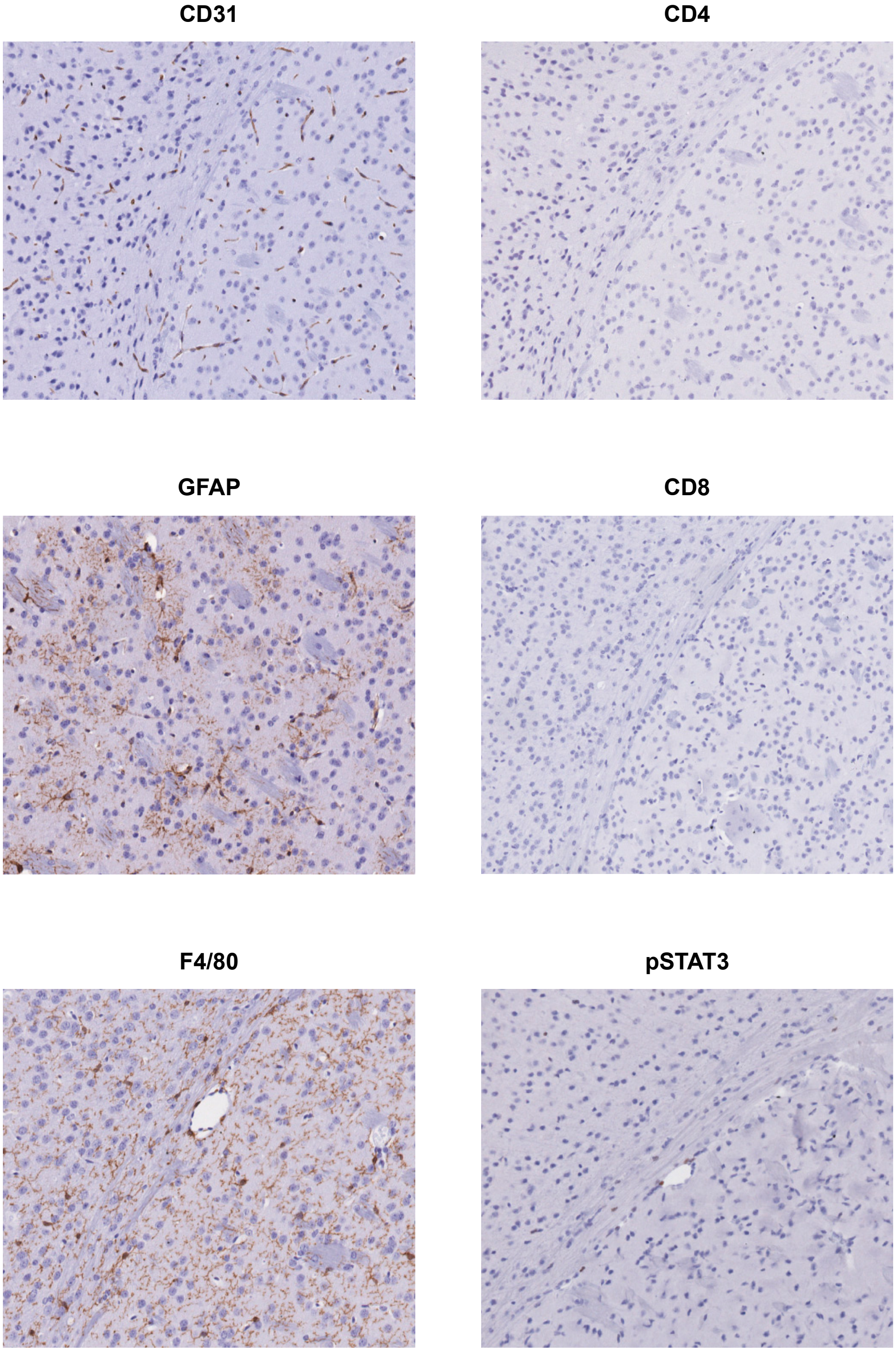

Supplementary figure S2: H-E-stained sections of brain tissue sample representative of RN pertaining to subject 11 (details provided in Table 1). The following areas have been demarcated and verified by two neuropathologists independently: radiation necrosis (black), gliosis (light blue) and residual tumor (red).

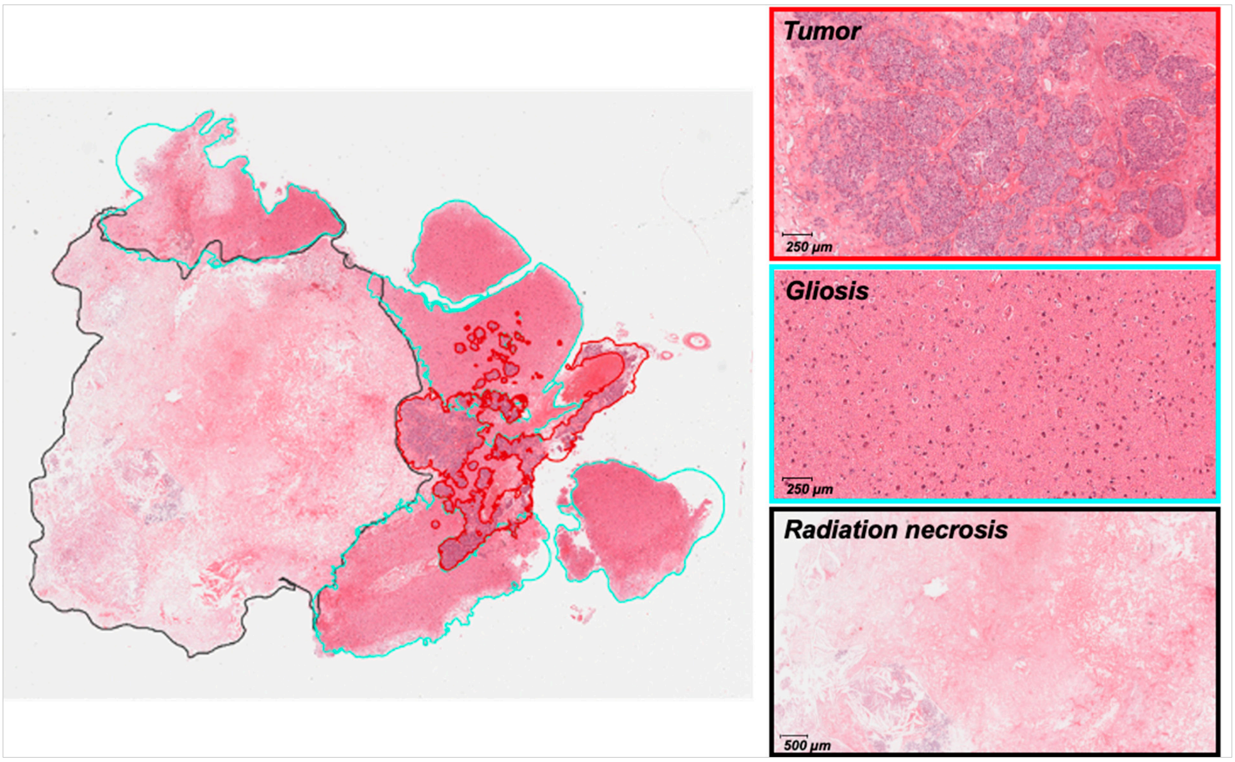

Supplement: Supplementary file 1 [file ijms-24-14219-s001.zip › ijms-2557916-supplementary.pdf]
